# Rupture of the Sclerotica in a Horse

**Published:** 1884-01

**Authors:** 

**Affiliations:** From Zeitshift für Augelheilkunde


### IV.—RUPTURE OF THE SCLEROTICA IN A HORSE.

BY DR. BERLIN.

*From Zeitshift für Augelheilkunde.*

A rather uncontrolable saddle horse was taken to the forge to be shod. On endeavoring to remove a fore shoe the horse reared and plunged and struck his head against a large round iron rod which ran in a horizontal direction and to which horses were generally fastened. The horse struck this object so hard as to cause him to fall and groan and the owner observed that blood and a transparent fluid was issuing from between the lids of the left eye.

I examined the horse on the evening of the same day and found a wound about the size of a quarter of a dollar in the middle of the superior eye lid, corresponding to the middle of the edge of the superior orbit. Between the lids exuded blood and some of the vitreous humor. The bulbus felt soft; on opening the lids a mass of blood and vitreous humor was found to fill the entire medial canthus. The entire anterior chamber was full of blood so that nothing could be seen of the posterior parts. The outflow of the vitreous humor without rupture of the cornea indicated that rupture of one of the sclerotica must have occurred, which was found to be situated on the inner side of the eye, out of which was pressed a portion of the retina. The sclerotica was not ruptured directly by the blow which the horse gave himself, but the accident was due to the sudden pressure thus exerted, the membrane bursting at its thinnest part, and at this particular point, because the pressure momentarily exerted by the blow served to support the other portion of the membranes of the eye, and the contents found its way out at the point where circumstances made the less resistance.

It was decided at once to remove the eye as healing of the wound could only be followed by shrinking of the bulbs after a long continued period of suppuration. Aside from this one had to consider the danger of sympathetic complications of the other eye.

Horse was cast. Narcosis, which necessarily must be very deep under such circumstances was produced with chloroform. The operator placed behind the head, leaning over it, one assistant by the neck behind the jaws and another before the head. The first assistant held the lids apart with appropriate instruments, and the third held the nictitans in a fixed position. Hooks of the largest size used in human surgery to take up the muscles are suitable to use in such operations on the eye of the horse, curved scissors of various sizes being also necessary.

Section of the muscles was found to be rather difficult on account of the contents of the eye oozing out of the wound during the operation, and the sequential collapse of the bulb. (Would it not be well in such cases to temporarily stitch up such a wound in order to retain the contents of the eye.—*Translator.*) In was found quite difficult for above reasons, to section the retractor muscles, not being able to attach it with a hook, but by manipulation I could feel its insertion with my finger and by this means and assistance of forceps, cut it properly. The optic nerve was then cut at once with scissors. The operation itself required seven minutes.

The treatment of the wound was very simple on account of the formation of the horse's head; bandages could not be so placed that they would not interfere with the movements of the jaws, so that I had previously prepared a piece of very fine sponge by cutting it so as to have approximately the form and size of the bulbus; after soaking it in carbolic acid solution, and before placing it in the cavity it was thoroughly wrung out and covered with a coating of powdered iodoform, the bleeding having previously stopped—it was but slight at the most—and the cavity thoroughly washed out. After three days the sponge was removed, being saturated with the secretion from the wounded surfaces, and replaced with another which had been treated in the same manner. The wound presented a clean granular surface as if freshly cut. The suppuration was very slight and on account of the viciousness of the horse it was decided not to replace the sponge after the second had been removed, which was on the sixth day. The wound was afterwards treated by being washed with a solution of boracic acid, 4.0 :200.0. An artificial eye of hard rubber was introduced four weeks after the operation and the horse took his place in the regiment to which he belonged. These artificial eyes work very nicely if they are only put in for the time the horse is in actual use and kept constantly clean as well as the cavity, which should be washed of every particle of dirt or dust when the eye is introduced.

